# Use of Autologous Leucocyte- and Platelet-Rich Plasma (L-PRP) in the Treatment of Aural Hematoma in Dogs

**DOI:** 10.3390/vetsci8090172

**Published:** 2021-08-28

**Authors:** Roberta Perego, Eva Spada, Elena Moneta, Luciana Baggiani, Daniela Proverbio

**Affiliations:** 1 Veterinary Transfusion Research Laboratory (REVLab), Department of Veterinary Medicine (DIMEVET), University of Milan, Via dell’Università 6, 26900 Lodi, LO, Italy; luciana.baggiani@unimi.it (L.B.); daniela.proverbio@unimi.it (D.P.); 2 Clinica Veterinaria Lainate, Via Nerviano 2, 20045 Lainate, MI, Italy; emoneta.vet@gmail.com

**Keywords:** aural hematoma, leucocyte- and platelet-rich plasma, dog, treatment

## Abstract

Leukocyte- and platelet-rich plasma (L-PRP) can accelerate the healing process by providing increased concentrations of platelet-derived growth factors. The objective of this study was to evaluate the clinical effect of L-PRP in the treatment of canine aural hematomas associated with otitis externa. Twenty mL of citrated whole blood was collected from each of the 17 dogs included and autologous L-PRP was produced. The aural hematoma was drained and 0.5–1 mL of L-PRP was injected. The dogs were examined weekly until 7 days after complete clinical healing. A final clinical follow-up was performed 6 weeks after the first treatment with L-PRP. If there was recurrence of the aural hematoma at the first follow-up, the treatment was repeated. In total, 2/17 cases were lost after the first follow-up. In 5/17 dogs, a short-term recurrence occurred. In 12/15 cases, complete clinical resolution was achieved with a single L-PRP application (Group A1) and in 3/15 with two treatments (Group A2). The mean time to complete clinical resolution was 16 ± 8.7 days (A1) and 23.3 ± 4 days (A2), respectively. No side effects were reported. The in situ administration of autologous L-PRP resulted in a complete resolution of the aural hematoma in all dogs that completed the clinical trial.

## 1. Introduction

Canine aural hematoma is a common pathological condition in clinical veterinary practice [1,2,3], although the true prevalence of the condition is unknown [4]. Aural hematomas are fluctuant swellings filled with serosanguinous fluid spontaneously accumulated under the skin on the concave surface of the pinna, leading to separation of the skin from the underlying cartilage [5]. The exact source of hemorrhage is not known but it is thought to come from branches of the great auricular arteries and veins within, under, or between the cartilage layers [5]. Although its etiology remains unknown, several pathogenic mechanisms have been proposed [6]. This condition most commonly occurs as a result of the shear forces created by violent head shaking or ear scratching and is associate with hypersensitive skin disease [2], otitis externa [7,8] and otocariasis [7]. Other postulated mechanisms are trauma [7,9,10], degenerative processes leading to splitting of the auricular cartilage [2] and inflammatory immune-mediated processes that damage cartilage [7]; the latter, however, was not confirmed by a more recent study [2]. To complicate matters, aural hematoma seems, in dogs, not to be a true hematoma but rather a serosanguinous effusion that develops over time [7]. 

Since the exact cause of aural hematoma is not known, numerous palliative procedures have been developed, with variably successful outcomes [6,11]. Although the chronicity of the hematoma should be taken into account, therapeutic objectives should identify and treat the underlying disease preventing recurrence [10], establish drainage, maintain tissue apposition and preserve the normal appearance of the ear to avoid excessive thickening and scar formation [3]. 

Results of minimally invasive medical treatment, by repeated fine-needle aspiration followed by local deposition of corticosteroids, are controversial [1,4,12,13]. The underlying principle of the more commonly used surgical treatment is drainage and compression of the cavity by through-and-through suturing of the pinna and compressive dressings [14]. Open drainage methods include surgical incision and placement of a Penrose drain with or without systemic corticosteroids [8], in combination with a variety of suturing techniques to eliminate dead space within the pinna [1,14]. Incisional techniques to improve drainage involve multiple biopsy punch holes, a longitudinal linear or S-shaped incision and excision of a small strip of skin to create a defect [1,3,14,15]. Techniques are described to relieve suture tension using stents [16] and X-ray film [17]. Alternatively, a closed, in-dwelling drain can be used [18]. Additional procedures using a carbon dioxide laser [19] and local proteolytic enzymes as an adjunct treatment are described [17]. Complications are common for each treatment modality and include recurrence, incomplete healing, pinna thickening and wrinkling, infections and necrosis of the pinna [15]. Stents may be associated with pinna infection and necrosis [15]. Postsurgical care often requires voluminous head- and ear-encompassing dressings. These are not only uncomfortable, sometimes requiring sedation to be applied or replaced, but may also aggravate infection and otitis. Asphyxiation due to improper dressing application has been reported [6]. If left untreated, aural hematoma may be followed by excessive scarring with cosmetically unacceptable deformations of the pinna, which may also predispose to otitis externa [15,20].

Platelet-rich plasma (PRP) is defined as a volume of autologous plasma with platelet concentration above baseline [21]. When activated, by adding for example calcium chloride, the platelets granule secrete a variety of cytokines and growth factors, including platelet-derived growth factor (PDGF), transforming growth factor-β (TGF-β), vascular endothelial growth factor-A (VEGF-A), basic fibroblast growth factor (bFGF), epidermal growth factor (EGF) and connective tissue growth factor (CTGF) [22,23,24]. These growth factors give PRP the properties of stimulation of cell proliferation, modulation of cellular differentiation, promotion of angiogenesis and extracellular matrix synthesis, decrease of inflammation and acceleration of healing processes [25,26,27,28,29]. Despite the wide use of PRP, there are no standardized protocols in PRP preparation, resulting in variations in composition, in particular, the leukocyte content [30]. The leukocytes in PRP can release high levels of pro-inflammatory cytokines, such as IL-1β and TNF-α, which increase the catabolism of extracellular matrix [31,32]. However, the presence of leukocytes in an injectable preparation of PRP, named leucocyte- and platelet-rich plasma (L-PRP), can increase the in situ production of growth factors, with an antibacterial activity and a potential analgesic effect [33,34].

In recent years, multiple effects of PRP have been documented in vivo and in vitro in human and veterinary medicine. In dogs, clinical studies are focused on orthopedics uses [35,36,37,38,39] and in dermatology for the treatment of wounds [40] or skin ulcers with extensive tissue loss [41,42,43], chronic ulcers [44], surgically induced wounds [45,46,47,48], alopecia [49] and for antimicrobial effects in infected skin wounds [50]. The results of these studies have been variable which may reflect the lack of standardization of the methods for obtaining the PRP. Therapeutic use of PRP has many advantages related to the safety of the blood component due to its autologous nature [21], the ease of collection, the rapid processing and the immediate availability. 

Therefore, the aim of this prospective clinical study was to evaluate the in vivo clinical effect of autologous L-PRP in the treatment of canine aural hematoma associated with otitis externa, following the excellent results obtained in our preliminary study in two dogs [51]. 

## 2. Materials and Methods

### 2.1. *Ethics Statement*

The study was carried out with client-owned dogs after approval by the Ethics Committee of the University of Milan (protocol number 13 January 2015) and with informed owner consent. 

### 2.2. Animals and Inclusion Criteria

Seventeen privately owned dogs comprising a variety of breeds, 8 males and 9 females, with an age range of 1–15 years (mean ± DS: 7.2 ± 3.6 years) (Table 1) affected by unilateral aural hematoma, due to unilateral or bilateral otitis externa, were included in the study. In all cases, there had been a history of previous fine-needle drainages, sometimes associated with local deposition of corticosteroid, with subsequent recurrence of aural hematoma. Inclusion criteria were aural hematoma secondary to pruritus and shaking of the head due to already diagnosed concurrent otitis externa, in dogs with a normal complete blood count, serum biochemistry and coagulation profile referred to the Department of Veterinary Medicine, University of Milan.

At the time of inclusion (D0) for each dog, signalment and medical history were collected and recorded. This included: type of otitis externa (unilateral or bilateral, acute or chronic), macroscopic clinical features of the ear canal and results of cytological examination of material in the ear canal at the time of diagnosis of otitis externa (when available). The therapeutic plan for the treatment of otitis externa and any previous therapeutic treatment carried out for the aural hematoma were recorded. Furthermore, local pruritus and/or shaking of the head was scored—0: absent, 1: mild, which is 1 to 5 on the PVAS scale, and 2: severe, which is more than 6 on the PVAS scale [52]—at the time of the appearance of aural hematoma. Finally, a complete clinical examination was carried out, photographs of the aural hematoma were taken and additional specific information was recorded: pinna affected (right or left), size (width, length, and thickness), presence or absence of scars detectable on palpation of the pinna and timing of onset (acute: ≤7 days; chronic: >7 days) (Table 1 and Table 2). 

Each dog was classified as compliant or non-compliant depending on its behavior in the clinical examination. During the study, targeted topical and/or systemic ongoing therapies for the treatment of otitis externa were allowed. No other therapy was added or allowed.

### 2.3. Autologous L-PRP Preparation

At D0, L-PRP was prepared using a semi-automated closed system previously evaluated by the same authors in dogs [53]. Briefly, the system is composed of a single use, sterile collection kit for blood sampling (CpunT 20 mL, Eltek S.p.A, Hone, AO, Italy), a dedicated centrifuge (Eltek Group, Hone, AO, Italy) and an automatic instrument for the separation of the L-PRP (Eltek Group, Hone, AO, Italy). The collection kit consists of a butterfly needle (19G) connected to a 20 mL syringe for blood aspiration, with an antibacterial filter on the access for anticoagulant addition (3 mL of 3.8% sodium citrate), and a 10 mL bag for the storage of L-PRP. For each subject, after adding 2 mL of anticoagulant, 20 mL of whole blood (WB) was collected from the cephalic vein. The collection port was closed with a clip, the anticoagulant access was opened, and 1 mL of 3.8% sodium citrate was added. 

After sample collection, the aspiration syringe connected to the 10 mL storage bag was centrifuged at 1200× *g* for 15 min, using the dedicated centrifuge with special adapters. At the end of centrifugation erythrocytes, buffy coat and supernatant plasma layers were clearly visible in the aspiration syringe. This was gently removed from the adapter and placed in the automatic separation instrument. The plastic clip between the syringe and storage bag was opened and the vertical movement of a plunger controlled by an optical reader isolated the supernatant plasma, the buffy-coat and the surface of the erythrocyte layer into the storage bag. After disposing of the aspiration syringe, the storage bag was centrifuged at 2000× *g* for 5 min, separating the pellet from the overlying platelet poor plasma (PPP). Finally, 75% of the supernatant PPP was removed with a sterile syringe through the appropriate perforable membrane, and the pellet was resuspended in 25% of the remaining PPP by gentle manual mixing. 

**Figure 1:**
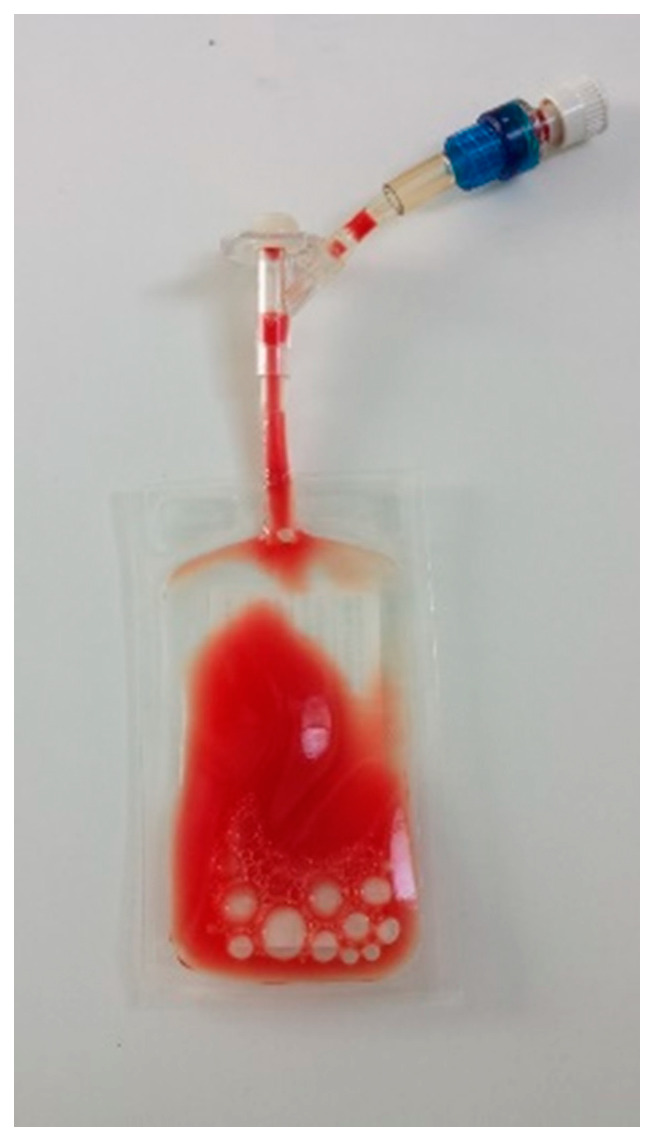
Final L-PRP bag ready for clinical use.

The leukocyte- and platelet-rich plasma (L-PRP) thus obtained (Figure 1) was collected through the perforable membrane with a sterile syringe. For each dog, leucocyte count (WBC/µL) and platelet count (PLT/µL) in an aliquot of WB and L-PRP were calculated by an automatic analyzer (Cell-Dyn 3500 analyzer, Abbott Diagnostics Europe). All samples were stored at room temperature on a laboratory blood rocker for 5 min before counts were performed.

### 2.4. L-PRP Application Protocol

**Figure 2:**
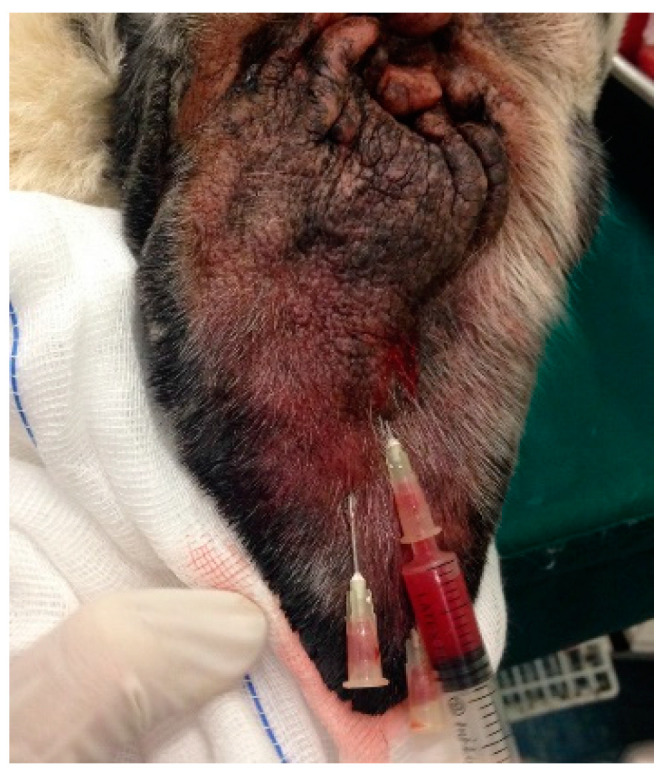
L-PRP application after drainage.

The ear pinna was clipped (if necessary) and disinfected with 10% povidone iodine solution (Betadine^®^). The fluid content of the hematoma was completely drained using one or more (depending on the presence of scars in the pinna) 20 G needles connected to a syringe and the volume removed was measured. Using the same needle/s left in situ, 0.5–1 mL, depending on how much obtained in each subject, of autologous L-PRP was then injected (Figure 2). The needles were removed, and the area was disinfected with 10% povidone iodine solution (Betadine^®^). Gentle pressure was applied to the hole with a sterile gauze for 2–3 min.

The dog was monitored for 15 min in the waiting room and then discharged without any bandage, drainage, containment tools or therapy other than that for the local and/or systemic treatment of concurrent otitis externa. 

### 2.5. Clinical Evaluation

After the L-PRP application, each dog was examined weekly until 7 days after complete clinical healing. Healing was recorded when the aural hematoma, together with associated otitis externa and related signs (pruritus and shaking of the head), completely disappeared. If there was recurrence of the aural hematoma (>50% of the size at D0) at the first follow-up, the L-PRP treatment was repeated following the same procedure. The presence of excessive scarring, longitudinal contractures and deformations of the pinna was assessed and recorded, and aural hematomas were photographed and measured at D0 and at each subsequent clinical follow-up. A final clinical follow-up was performed 6 weeks after D0 (D42). A telephone follow-up was performed 6 months after D0 for each dog.

### 2.6. Statistical Analysis

The normal distribution of parametric data was calculated using the D’Agostino-Pearson test and only PLT values in whole blood were found to be nonnormally distributed. Results are presented as mean ± standard deviation. The statistical differences between median values of PLT and WBC on WB and L-PRP were compared using Wilcoxon rank sum test or paired *t*-test depending on data distribution. The increase in platelet and leucocyte concentration in L-PRP over whole blood baseline values was calculated using the following equation: platelet or leucocyte count in L-PRP platelet or leucocyte count in WB/platelet or leucocyte count in WB × 100. The possible association between the clinical variables timing of onset (acute: ≤7 days; chronic: >7 days), presence/absence of scars detectable on palpation of the pinna at D0, quantity of drained fluid (<10 mL or ≥10 mL) at D0, number of L-PRP applications, type of otitis, score of pruritus/shaking of the head at D0 and time to complete clinical healing were evaluated using a Mann–Whitney test or an independent sample *t*-test depending on the data distribution. For all tests, significance was set at *p* < 0.05. Statistical analyses were performed using commercial software (MedCalc Software version 11.5.1 Mariakerke, Belgium).

## 3. Results

### 3.1. L-PRP Analysis

L-PRP obtained and subsequently injected had a mean PLT concentration of 1033 ± 573 × 10^3^/μL (minimum value: 308 × 10^3^/μL, maximum value: 2241 × 10^3^/μL) with a 347% mean increase compared to whole blood. The mean leukocyte concentration in the PRP was 15,078 ± 3109 WBC/µL, corresponding to a mean percentage increase of 92% compared to whole blood. The comparison between platelet and leukocyte concentrations in WB and L-PRP showed a statistically significant increase for both variables (Table 3).

### 3.2. Clinical Study Results

Only one dog was classified as non-compliant and, in this dog, L-PRP administration was performed under general anesthesia, with the owner’s informed consent. 

At D0, for the 17 dogs included, the aural hematoma had been present for a mean of 19.2 ± 12.4 days and the mean volume of the drained fluid content was 23.3 ± 30.4 mL. Two dogs were withdrawn from the study after the first follow-up since the owners opted for surgical treatment following the recurrence of the aural hematoma after the first L-PRP treatment. Thus, 15 dogs completed the study (Table 2). 

In 12 dogs, clinical healing occurred following a single L-PRP application (Group A1), and in 3/15 after two applications 7 days apart (Group A2): the mean time to clinical healing was 16 ± 8.7 and 23.3 ± 4 days, respectively. (Table 2). There was no statistical correlation between clinical variables and healing (Table 4).

**Figure 3:**
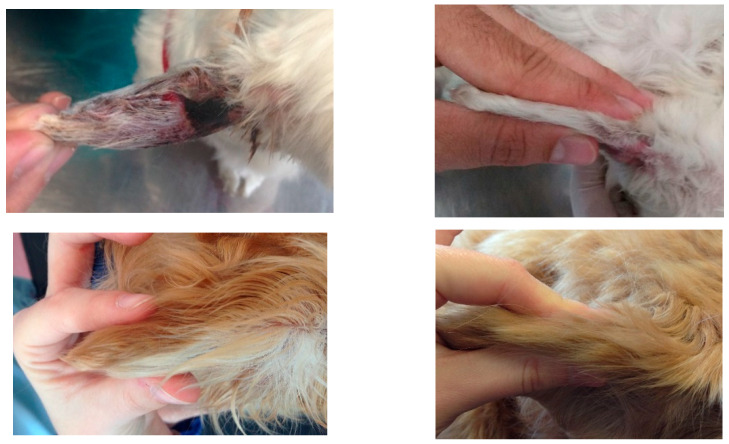

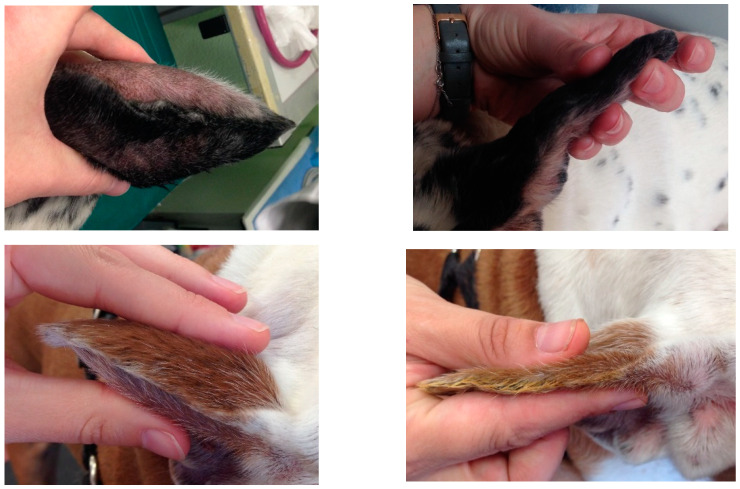
Macroscopic images of the pinna of four cases (dog 3, 10, 12 and 13) at D0 (**left**) and D42 (**right**).

No side effects were recorded. No clinical signs, such as erythema, pain or other clinical alteration indicating an inflammatory state, were found at the injection site in the treated dogs. No dog had excessive scarring, longitudinal contractures or deformation of the treated pinna (Figure 3). No recurrences was seen in any dog at the 6-week follow-up. The telephone follow-up scheduled 6 months after D0 was completed in 12 of the 15 subjects and none had relapsed. 

## 4. Discussion

The uncertain etiopathogenesis of canine aural hematoma and lack of evidence comparing the efficacy of available treatments has resulted in the continued use of several techniques. The most popular initial treatment reported in a UK survey is needle drainage with or without the concurrent use of local corticosteroids [4]. Given the frequent recurrence with conservative-medical treatment of aural hematoma, surgical management is commonly employed [6]. The surgical procedures classically recommended [14] are often uncomfortable for the patient and may result in complications such as recurrence, cosmetically unacceptable deformations of the pinna, skin irritation and infection. The surgical technique of continuous vacuum drainage described in a recent study [6] seems to provide good patient comfort, tolerance of drains, absence of dressings and good cosmetic outcome. However, this does require general anesthesia, access to appropriate materials and experience to perform. Furthermore, the drainage equipment must be maintained for 2–3 weeks in combination with an Elizabethan collar and, in some cases, sedation is required for drainage removal [6].

In this prospective clinical study, we tested the clinical effect of L-PRP in treatment of aural hematoma based on hemostatic, anti-inflammatory and angiogenic effect of autologous L-PRP demonstrated by several previous studies in dogs [47,48,54]. Furthermore, recently, a human review to evaluate the efficacy of PRP in reducing post-operative split-thickness skin graft loss and hematoma formation showed that PRP decreased the odds of hematoma formation by 79% [55].

This is the first report describing the use of L-PRP for the treatment of aural hematoma in dogs and demonstrates that autologous L-PRP appears to be an effective, safe and easy-to-perform method for treating this condition in dogs. 

The L-PRP treatment performed in our study resulted in a complete clinical healing in all 15 subjects who completed the clinical trial, without excessive scarring, longitudinal contractures and deformations of the treated pinna or side effects in any dog. In the literature, the preservation of the appearance of the pinna following the treatment of aural hematoma is considered one of the objectives of the medical or surgical therapy [1,15,20]. None of the 15 clinical cases that completed the trial had a curling of the ear pinna. This complication is not only esthetically unacceptable to owners, but can increase the risk of further otitis externa, after resolution of aural hematomas [15]. The percentage of resolution of aural hematoma with autologous L-PRP treatment used in this study is comparable to previous studies with other medical and surgical techniques [6,7,56].

It is interesting to note that in two dogs in our study which achieved complete recovery with L-PRP treatment, cortisone injection therapy had previously been used in loco with no effect. Injectable in situ corticosteroid therapy is one of the treatments described for the resolution of aural hematoma, for its anti-inflammatory effect and suppression of the production of fluid production [12,57]. It was not possible to compare the efficacy of L-PRP and corticosteroid therapy for aural hematoma treatment in this study due to the small number of cases treated with both therapies, and because type and dose of corticosteroid used was not known.

The mean healing time with L-PRP treatment was 17 ± 13 days, which is longer than the healing time after a surgical approach (where removal of sutures occurs around 9 to 12 days after surgery) [58]. However, the great advantage of treatment with L-PRP compared to a surgical approach is the ease of execution, which requires no specialized operator skills and, except in rare cases, there is no need for sedation of the patient. Another big advantage of the L-PRP approach is the total absence of drains, bandages or other containment tools after treatment, which can cause significant discomfort to the patient after the surgical approach [6]. Owner compliance, which is essential to avoid serious complications post-surgery [6] is not necessary post-treatment with L-PRP, as the only home care required is therapy for the underlying otitis. This aspect makes L-PRP treatment a valid option when the owner is thought to be non-compliant.

In 5/17 (30%) dogs in this study, there was early aural hematoma recurrence, 7 days after treatment. Three of these dogs underwent a second L-PRP treatment, resulting in the complete resolution of the aural hematoma in the following days. In the other two cases, the owners opted instead for a surgical approach and abandoned the clinical trial. In the other 10 dogs, at 7 days, the aural hematoma was clearly less than 50% of the initial size or completely absent. No long-term relapse was reported at the telephone follow-up carried out 6 months after the start of the trial. The long-term relapse prevention effect highlighted by this study agrees with the data emerging from a human study on the efficacy of autologous PRP for the treatment of muscle rupture with hematoma, in which the researchers found a lower rate of recurrent hematoma in the PRP group than in the control group [59].

The 30% short-term recurrence rate after the first L-PRP treatment is lower than that recently reported with the most common medical treatment used for aural hematoma (51% for needle drainage with local deposition of corticosteroids) and slightly higher than the upper limit reported for surgical approach (25%) [4].

No side effects were reported after the administration of L-PRP. This is probably due to the autologous origin of the product, which, in our study, was produced in sterile conditions with a semi-automated closed system and used immediately after production. This procedure prevented any contamination or bacterial proliferation in the blood component, which could lead to clinical complications and affect clinical efficacy [60].

Patient management was easy during all phases of treatment, from blood sampling for the preparation of L-PRP to post-therapeutic management carried out by the owners at home. Only 1 out of 17 patients, which was a very anxious dog and intolerant to any medical approach, needed to be treated under general anesthesia. The possibility of carrying out the treatment without general anesthesia is an important advantage of the therapy with L-PRP over the surgical approach, particularly in old or critically ill patients.

The average platelet concentration in L-PRP in this study was 1033 ± 573 × 10^3^/μL, with a 347% mean increase compared to whole blood. This, according to the literature, defines the concentrate obtained as a therapeutic PRP suitable for clinical use [25].

The mean leukocyte concentration in the PRP was 15,078 ± 3109 WBC/µL, corresponding to a mean percentage increase of 92% compared to whole blood. The presence of leukocytes in platelet concentrate is a subject of controversy in the literature. Some researchers have argued that the presence of leukocytes in the platelet concentrate intended for therapeutic application is a negative aspect with harmful effects on damaged tissue, given the leukocyte pro-inflammatory activity [61,62]. Conversely, other researchers have demonstrated how the presence of leukocytes in injectable preparations of PRP provides a useful increase of in situ production of growth factors [63], which results in release of pain mediators [64,65] and ensures a natural anti-infectious action [65]. In our study, no clinical inflammatory reactions were observed following the L-PRP treatment.

In our study, the timing of complete clinical healing was not associated with any of the variables. However, the greater quantity of fluid drained and the need for two applications of L-PRP significantly increased healing times. Our data in fact suggest that very high volumes of fluid drained at D0 (and in particular a volume > 80 mL) are probably associated with the need for a second application of L-PRP. In fact, 100% of the subjects in which a volume equal to or greater than 80 mL was drained at D0 (3 subjects), relapsed at the first follow-up. Unfortunately, only one of the three dogs continued the trial with a second L-PRP application and showed complete clinical healing at D30. In the other two dogs, the owners abandoned the clinical trial, opting for a surgical resolution; thus, it was not possible to statistically evaluate this association. A possible hypothesis for the association between high volumes of fluid drained and the need for a second L-PRP treatment is that when a high volume of fluid has collected in the pinna, there is a large area of detachment of the auricular surfaces and, at the same time, compression exerted by blood collection can induce cell necrosis by crushing and/or stretching and delaying healing. 

The lack of a case-control study design is a limitation in this study, as there are no data on what would have happened to the aural hematomas either without any treatment or with a different medical or surgical treatment. It should be noted, however, that all subjects treated with L-PRP had previously undergone needle drainage with or without local corticosteroid injection, with recurrence.

Other limitations of our study include the relatively low number of dogs and the inclusion of only dogs that had been previously treated with a classical medical approach, which may have affected the outcome of the L-PRP treatment.

Another limitation may be the simultaneous use of local corticosteroids instilled in the ear canal of all subjects, associated with oral prednisolone in two subjects, a therapy that may also have promoted aural hematoma resolution. It should be noted, however, that these therapies were already in place during the previous hematoma treatment and none of the hematomas had resolved at that time.

We also did not measure the final thickness of treated pinna, comparing the measurements with the unaffected pinna. Instead, only the measurements of the aural hematoma over time, its disappearance and the gross absence of excessive scarring, longitudinal contractures and deformations were monitored. The lack of these data, associated with the absence of a histological evaluation of the pinna before and after L-PRP treatment, made the assessment of cosmetic outcome rather subjective. It should be emphasized, however, that in previous clinical studies that have evaluated other methods treatment for aural hematoma, healing was also assessed in a similar way, i.e., by evaluation of the reduction in fluid collection in the pinna [6,13,20]. Biopsy of tissue to demonstrate microscopic healing could not be justified.

Future studies are needed to evaluate more objectively the cosmetic results and to investigate the correlations between chronicity of aural hematoma and healing times or clinical relapse after treatment with L-PRP and to evaluate the possible correlations with macroscopic and hematological characteristics of the drained fluid.

## 5. Conclusions

In conclusion, this study shows that the in situ administration of autologous L-PRP is a safe and well tolerated treatment for aural hematoma secondary to otitis externa in dogs, leading to a complete and long-term resolution of the disease.

## Figures and Tables

**Table 1:** Descriptive data for each dog. The dogs marked with (*) did not complete the clinical trial.

Dogs	Breed	Gender	Age (Years)	Weight (kg)	Type of OE	Cytology Results at the Time of Diagnosis of OE	Clinical Features of Ear Canal at the Time of Diagnosis of OE	Ongoing Therapy for OE	**Score of Pruritus/Shaking**	**Number of Previous Drainages of Hematoma**
1	Rhodesian Ridgeback	F	7	30	Chronic, unilateral	Cocci	Erythema, mild stenosis and discharge	Local drops with miconazole, prednisolone, polymyxin B	2	2 + GCs
2	Rhodesian Ridgeback	F	8	31	Acute, unilateral	Cocci	Not available	Local drops with miconazole, prednisolone, polymyxin B	1	1
3	Mixed breed	M	4	28	Chronic, unilateral	Cocci + Malassezia spp.	Erythema, mild stenosis and abundant ear wax	Local drops with gentamicin, econazole, tetracaine, flumetasone	2	2
4	German shepherd	M	3	36	Chronic, bilateral	Malassezia spp.	Mild stenosis and abundant ear wax	Local drops with climbazole, ceramide, 18-beta- glycyrrhetinic acid, palmitoylethanolamide	1	1
5 *	Mixed breed	NF	15	21	Chronic, bilateral	Rods	Swelling, mild stenosis, ulcerations and discharge	Local drops with marbofloxacin, clotrimazole, dexamethasone	1	2 + GCs
6	Staffordshire Bull Terrier	NF	12	19	Acute, unilateral	Not available	Not available	Local drops with terbinafine, florfenicol, betamethasone	2	1
7	Boxer	NF	7	27	Acute, bilateral	Cocci + Malassezia spp.	Erythema and abundant ear wax	Local drops with terbinafine, florfenicol, betamethasone	2	1
8	Boxer	NF	6	30	Acute, bilateral	Cocci	Erythema and discharge	Local drops with miconazole, prednisolone, polymyxin B	1	1
9 *	Beagle	M	9	17	Chronic, bilateral	Rods	Not available	Local drops with orbifloxacin, mometasone, posaconazole	1	2
10	Bulldog Inglese	F	6	24	Chronic, bilateral	Cocci and rods	Erythema, swelling, moderate stenosis	Local drops with gentamicin, miconazole, hydrocortisone aceponate + oral prednisolone at 0.5 mg/kg/day for first 10 days	1	1
11	Mixed breed	M	10	22	Chronic, bilateral	Rods	Erythema, swelling, discharge, severe stenosis	Oral enrofloxacin 5 mg/kg/day + oral prednisolone at 0.5 mg/kg/day	2	2
12	Golden Retriever	F	8	30	Chronic, bilateral	Cocci and rods	Not available	Local drops with marbofloxacin, clotrimazole, dexamethasone	1	1
13	Mixed breed°	NF	8	23	Chronic bilateral	Cocci and Malassezia spp.	Erythema, mild stenosis and abundant ear wax	Local drops with gentamicin, econazole, tetracaine, flumetasone	1	1
14	English Foxhound Terrier	NM	12	26	Chronic, bilateral	Cocci	Erythema, mild stenosis and swelling	Local drops with miconazole, prednisolone, polymyxin B	2	2
15	Australian shepherd	M	1	33	Chronic, bilateral	Not available	Not available	Local drops with climbazole, ceramide, 18-beta- glycyrrhetinic acid, palmitoylethanolamide	2	1
16	Labrador retriever	M	6	38	Chronic, bilateral	Rods + Malassezia spp.	Erythema, abundant ear wax, moderate stenosis	Local drops with marbofloxacin, clotrimazole, dexamethasone + oral marbofloxacin at 2 mg/kg/day	1	2 + GCs
17	Labrador retriever	M	4	42	Acute, bilateral	Cocci	Erythema, discharge	Local drops with miconazole, prednisolone, polymyxin B	2	1

Legend: M, male; F, female; NF, neutered female; NM, neutered male, GCs, glucocorticoids; OE, otitis externa. The dog marked with (°) was the only dog classified as non-compliant.

**Table 3:** Mean platelet and leukocyte mean concentration in WB and L-PRP.

	Sample	Mean ± DS	Mean Increase (%)	*p*
**WBC (cells/μL)**	WB	8076 ± 1689		
	L-PRP	15,078 ± 3109	92	**<0.0001**
**PLT (10^3^/μL)**	WB	229 ± 66		
	L-PRP	1033 ± 573	347	**<0.0001**

**Table 4:** Correlation between clinical variables and mean healing times in 15 dogs that completed the clinical trial.

	Number of Dogs	Mean Clinical Healing Time (days)	*p*
**Number of L-PRP applications**			
one	12	16 ± 8.7	0.07
two	3	23.3 ± 4	
**Timing of onset**			
acute (≤7 days)	5	16.8 ± 10.6	0.85
chronic (>7 days)	10	16 ± 6.7	
**Quantity of drained fluid at D0**			
<10 mL	7	13 ± 4.8	0.07
≥10 mL	8	20 ± 8.5	
**Presence of scars on the pinna at D0**			
yes	3	21 ± 7	0.26
no	12	16 ± 8.8	
**Type of otitis externa**			
unilateral	4	14 ± 9.9	0.30
bilateral	11	19 ± 6.7	
**Score of pruritus/shaking at D0**			
1	7	17 ± 6.8	0.86
2	8	18 ± 7.9	

**Table 2:** Descriptive data of each aural hematoma at D0. The dogs marked with (*) did not complete the clinical trial.

Dog	Affected Pinna	Timing of Onset of Hematoma (days)	Timing of Recurrence after Initial Treatment (days)	Size ad D0 (Length × Width × Thickness in Centimeters)	Scars at D0	mL Drained at D0	Number of L-PRP Treatment	Volume of L-PRP Administered (mL)	mL Drained at D7	Day of Clinical Healing
1	Right	21	5 and 15	4.5 × 5 × 2.5	No	8	1	1	-	14
2	Right	7	3	2 × 2 × 1	No	0.5	1	1	-	7
3	Right	15	6 and 10	7 × 4 × 3	No	10	1	0.5	-	7
4	Right	15	4	4 × 3 × 2	No	5	1	1	-	14
5 *	Left	40	10 and 21	10 × 5 × 5	Yes	85	1	0.5	CR	-
6	Right	7	Not available	7 × 7 × 2	No	10.5	1	0.5	-	28
7	Left	3	2	6 × 4 × 4	No	17	1	1	-	28
8	Right	4	3	4 × 2 × 1	No	10	1	1	-	14
9 *	Right	30	7 and 15	9 × 7 × 5	No	80	1	0.5	CR	-
10	Right	21	Not available	3 × 1 × 1	No	3	1	0.5	-	14
11	Right	30	5 and 19	3.5 × 4 × 2	No	7	1	0.5	-	14
12	Right	21	15	4 × 5 × 2.5	Yes	10	2	1 and 1	8	28
13	Left	15	7	10 × 6 × 8	Yes	80	2	1 and 1	55	21
14	Right	45	Not available	10 × 9 × 3.5	Yes	15	1	0.5	-	14
15	Right	15	8	8 × 4 × 2	No	4	2	1 and 1	4	21
16	Left	30	8 and 22	5 × 3 × 4	No	10	1	1	-	21
17	Right	7	4	3 × 6 × 5	No	5	1	0.5	-	7

Legend: CR, complete recurrence (the actual measurement in ml was not possible because the owner opted for surgical treatment).

## Data Availability

The raw data supporting the conclusions of this article will be made available by the authors.
